# Cold Plasma as a Novel Pretreatment to Improve the Drying Kinetics and Quality of Green Peas

**DOI:** 10.3390/foods14010084

**Published:** 2025-01-01

**Authors:** Jun-Wen Bai, Dan-Dan Li, Reziwanguli Abulaiti, Manqian Wang, Xiaozhi Wu, Zhenwei Feng, Yutong Zhu, Jianrong Cai

**Affiliations:** School of Food and Biological Engineering, Jiangsu University, Zhenjiang 212013, China; bjw@ujs.edu.cn (J.-W.B.); lidandan_916@163.com (D.-D.L.); 3210910098@stmail.ujs.edu.cn (R.A.); 13693913107@163.com (M.W.); xiaozhi_wu03@163.com (X.W.); fzw200106@163.com (Z.F.); zyt173664@163.com (Y.Z.)

**Keywords:** green peas, cold plasma, drying kinetics, microstructure, quality

## Abstract

Green peas, with their high moisture content, require effective drying techniques to extend shelf life while preserving quality. Traditional drying methods face challenges due to the dense structure of the seed coat and wax layer, which limits moisture migration. This study investigates cold plasma (CP) pretreatment as a novel approach to enhance drying kinetics and maintain the quality attributes of green peas. The results showed that CP treatment significantly improves drying efficiency by modifying the pea epidermis microstructure, reducing drying time by up to 18.18%. The moisture effective diffusivity coefficients (*D_eff_*) for untreated and CP-pretreated green peas were calculated to range from 5.9629 to 9.9172 × 10^−10^ m^2^·s^−1^, with CP pretreatment increasing *D_eff_* by up to 66.31% compared to the untreated group. Optimal CP parameters (90 s, 750 Hz frequency, 70% duty cycle) were found to improve the rehydration ratio, preserve color, and increase total phenolic content (TPC) by 24.06%, while enhancing antioxidant activity by 29.64%. Microstructural changes, including pore formation and increased surface roughness, as observed through scanning electron microscopy (SEM), partially explain the enhanced moisture diffusion, improved rehydration, and alterations in nutrient content. These findings underscore the potential of CP technology as a non-thermal, eco-friendly pretreatment for drying agricultural products, with broad applications in food preservation and quality enhancement.

## 1. Introduction

Green peas (*Pisum sativum L.*) are leguminous plants cultivated extensively worldwide. Their palatable taste and abundant nutritional profile have contributed to their widespread use in diverse culinary applications [1,2]. Fresh green peas have a high moisture content, resulting in a short shelf life of only a few days at room temperature. The most common processing methods for peas include freezing, canning, and drying [3,4]. Drying processes, such as hot air drying, microwave drying, infrared drying, and vacuum freeze drying, are commonly used to reduce the bulk and weight of products [5,6]. These drying operations effectively eliminate the need for a cold chain during subsequent storage and transportation, making them highly applicable in the food industry [7].

The surface of the pea is encased in a seed coat that serves as a protective layer, which naturally shields the embryo and acts as a barrier against external threats [8,9]. This seed coat is rich in lignin, and its tightly packed cells create a dense barrier that restricts the movement of water and gases [10]. Additionally, the outer surface of the seed coat is covered by a waxy cuticle, which further reduces permeability and complicates moisture escape during drying [11,12]. As a result, the pea’s naturally protective seed coat structure, while providing resilience against external damage, also acts as a primary obstacle to moisture migration. This barrier prolongs drying time and complicates the preservation of product quality. Consequently, pretreatment techniques aimed at modifying the seed coat structure are necessary to address this challenge.

Researchers have explored various pretreatment methods to enhance moisture transfer through the seed coat of peas, aiming to reduce drying time and energy consumption. More and Tayade [13] used blanching and pricking techniques to accelerate drying and improve the rehydration ratio. Prakash Pandey et al. [14] applied a blanching pretreatment with hot water and citric acid, demonstrating that this method not only lowered drying costs but also preserved the quality of green peas. Similarly, studies by Yang et al. [15] and Yu et al. [16] found that ultrasound treatment enhanced drying kinetics in pea seeds, reducing drying time and increasing the diffusion coefficient. Baier, Bußler, and Knorr [17] showed that high pressure and pulsed electric fields modified the cellular structure of pea tissue, further improving drying and rehydration efficiency. These findings underscore the effectiveness of diverse pretreatment methods in enhancing both drying and rehydration efficiency for green peas. As researchers continue to explore innovative techniques, recent advances in cold plasma pretreatment show great potential for improving the drying process of agricultural products, offering new avenues for enhancing product quality and efficiency.

Cold plasma (CP) is emerging as a promising alternative technology for food processing [18]. CP is an ionized gas generated under atmospheric or low-pressure conditions, consisting of a mixture of reactive species, including high-energy electrons, positively and negatively charged ions, free radicals, and neutral molecules [19]. These reactive species interact with the surface of the treated material, inducing a range of physical, chemical, and biological changes, such as surface etching, oxidation, and the alteration of surface hydrophilicity [20]. As a non-thermal processing method, CP can be applied as a pretreatment in food drying. Compared to traditional methods like thermal, chemical, and irradiation treatments, CP offers significant advantages [21]. Recent studies demonstrate that CP pretreatment enhances drying efficiency and improves nutrient and flavor retention in dried foods. For example, CP-pretreated apple slices exhibited improved drying kinetics and higher drying efficiency, along with significantly elevated levels of antioxidant activity, phenolics, flavonoids, and ascorbic acid compared to untreated samples [22]. CP pretreatment also reduced drying time for mango slices by 20%, with samples treated for 5 min displaying higher total flavanol and phenolic contents [23]. In tobacco leaves, cold atmospheric plasma pretreatment shortened natural drying times by 37.7% for leaf veins and 19.5% for the entire leaf, while also enhancing the carbohydrate-to-protein ratio and reducing nicotine content [24]. Zhou et al. [25] found that optimal CP treatment could reduce drying time by up to 50% and increase rehydration rates by 7–16%. Additionally, CP pretreatment has been applied successfully to shiitake mushrooms [26], grapes [27], chili peppers [28], and garlic slices [29]. Consequently, CP technology has become an innovative pretreatment technique for drying agricultural products, with considerable potential for application. However, there has been limited research on the use of CP technology as a pretreatment for drying green peas, and the effects of treatment parameters on drying time and quality remain areas for further study.

This study aims to investigate the effects of CP treatment time, frequency, and duty cycle on drying kinetics, as well as to examine how CP pretreatment influences both the internal and external quality characteristics of dried peas, including color, rehydration ratio (RR), total phenolic content (TPC), and antioxidant activity. Additionally, scanning electron microscopy (SEM) will be employed to observe the impact of pretreatment on the microstructure of the pea epidermis, allowing for a more detailed analysis of how CP affects drying efficiency and overall product quality.

## 2. Materials and Methods

**Figure 1:**
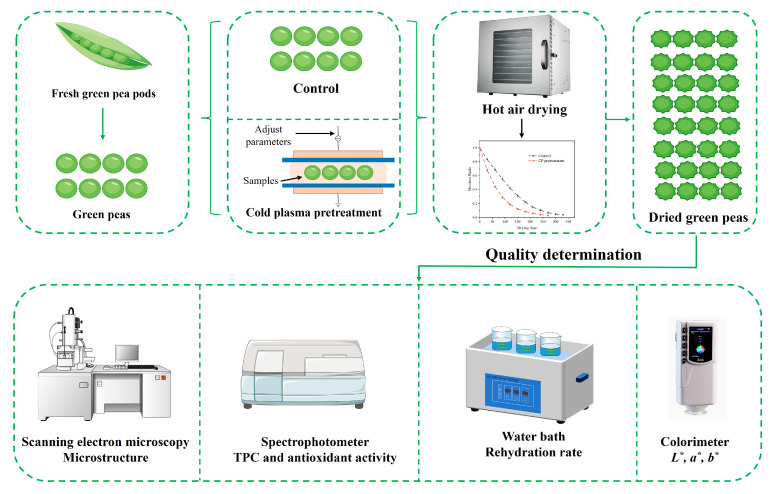
Illustrative flowchart of sample preparation, hot air drying, and quality determination of green peas.

The illustrative flowchart of sample preparation, hot air drying, and quality determination of green peas is shown in Figure 1.

### 2.1. Raw Samples

Fresh green pea pods were purchased from Jimailong Supermarket near Jiangsu University. After shelling, only peas with intact surfaces, plumpness, and uniform size were selected for the experiment. The average radius of the green pea samples was 0.45 × 10^−2^ m. The initial moisture content was measured at 77.51 ± 1.83% (wet basis, w.b.) using a quick moisture analyzer (DHS-16A, Shanghai Lichen Instrument Technology Co., Ltd., Shanghai, China). The green peas were stored in hermetically sealed bags at 4 °C until the experiment began.

### 2.2. Cold Plasma Pretreatment

The cold plasma treatment device used in this experiment was a dielectric barrier discharge (DBD) system, which included a high-voltage power supply (CTP-2000K, Suman Plasma Technology Co., Nanjing, China), voltage regulator (TDGC2-1, Chint Electric Company, Wenzhou, China), DBD reactor, and electrical system. The DBD reactor comprised two metal electrodes (55 mm in diameter, 8 mm thick) and a quartz dielectric. Pea samples were placed in the reactor, where an electrode voltage of 20 kV and an electrode gap of 12 mm were applied for treatment. Based on preliminary experiments, the plasma treatment parameters—treatment times (30, 60, 90, and 120 s), excitation frequencies (250, 500, 750, and 1000 Hz), and duty cycles (30%, 50%, 70%, and 90%)—were selected, as each factor influenced the degree of etching on the seed coat. Green pea samples of equal weight (approximately 15.0 g) were treated in the DBD reactor under these conditions, with all treatments performed in triplicate.

### 2.3. Hot-Air Drying Experiment

The CP-pretreated green peas were evenly spread in a single layer on a stainless-steel mesh bracket. The samples were then dried in a hot air-drying drier developed by Jiangsu University [30]. Drying was carried out at a temperature of 60 °C, an air velocity of 3 m/s, and a relative humidity of approximately 15%. Samples were removed from the drying chamber every 30 min for weighing, with a measurement accuracy of ±0.01 g. Drying was stopped when the green peas reached a final moisture content of 12.00% (wet basis).

### 2.4. Drying Kinetics

The moisture ratio was calculated by Equations (1) and (2) [31].

(1) $$MR=\frac{M_{t}-M_{e}}{M_{0}-M_{e}}$$

where $M_{0}$ is the initial dry basis moisture content, g/g; $M_{t}$ is the dry basis moisture content at the drying time *t*, g/g; MR is the moisture ratio; $M_{e}$ is the equilibrium moisture content, g/g. The equilibrium moisture content, $M_{e}$, is much smaller than $M_{t}$ and $M_{0}$ and can generally be ignored [32]. Therefore, the calculation of MR can be simplified as:

(2) $$\;MR=\frac{M_{t}}{M_{0}}$$

### 2.5. Determination of Moisture Effective Diffusivity (D_eff_)

Drying occurs mostly in the falling rate period, and moisture transfer during drying is controlled by internal diffusion. The Fick’s second law of diffusion equation has been widely used to describe the drying process during the falling rate period for agricultural materials. The solution of the diffusion equation, in spherical coordinates, and supposed uniform initial moisture distribution, constant diffusivity, negligible external resistance, and shrinkage, is expressed as follows [33]:

(3) $$MR=\frac{6}{\pi^{2}}\sum_{n=1}^{\infty }\frac{1}{n^{2}}exp\left({-n}^{2}\pi^{2}\frac{D_{eff}t}{r^{2}}\right)$$

where *D_eff_* is the effective moisture diffusivity (m^2^/s); *r* is radius of the green pea samples, with 0.45 × 10^−2^ m as its value; *t* is the drying time expressed in second (s); and *n* is a positive integer. For long time drying times, Equation (4) can be further simplified to a limiting form of the diffusion equation and expressed in a logarithmic form (Equation (4)):

(4) $$lnMR=ln\left(\frac{6}{\pi^{2}}\right)-\left(\frac{\pi^{2}D_{eff}}{r^{2}}\right)t$$

The effective moisture diffusivity is obtained by plotting the experimental drying data in terms of *lnMR* versus time (min). From Equation (5), a plot of *lnMR* versus time gives a straight line with a slope of (*k*), in which

(5) $$k=-\frac{\pi^{2}D_{eff}}{r^{2}}$$

### 2.6. Color Measurement

The color of fresh and dried green peas was analyzed using a colorimeter (SC-10, Shenzhen Sanenchi Technology Co., Ltd., Shenzhen, China) to determine the color coordinates L^*^ (dark–light value, 0~100), a^*^ (red–green value, −60~60), and b^*^ (blue–yellow value, −60~60) [34]. Each color parameter test was replicated six times, and the average results were calculated. In addition, the total color difference (Δ*E*) was calculated by Equation (6).

(6) $$∆E=\sqrt{{\left({L_{0}}^{*}-L^{*}\right)}^{2}+{\left({a_{0}}^{*}-a^{*}\right)}^{2}+{\left({b_{0}}^{*}-b^{*}\right)}^{2}}$$

where, ${L_{0}}^{*}$, ${a_{0}}^{*}$, ${b_{0}}^{*}$ are the color parameters of the fresh green peas, and $L^{*}$, $a^{*}$, $b^{*}$ are the color parameters of the pretreated dried green peas.

### 2.7. Rehydration Ratio (RR)

The rehydration ratio (RR) was determined as an indicator of the sample’s ability to absorb water and regain its original structure and texture. It provides insights into the effects of pretreatment and drying conditions on the cellular integrity and quality of dried products [35]. Dried green pea samples (approximately 5 g) were weighed and then immersed in distilled water at 95 °C for 60 min. After the specified rehydration time, the samples were carefully removed, surface water was gently blotted with filter paper to remove excess moisture, and samples were reweighed. The rehydration ratio (*RR*) was calculated as the ratio of the weight of the rehydrated sample (*Wr*) to the initial weight of the dried sample (*W*_0_), as shown in the Equation (7):

(7) $$\mathrm{RR}=\frac{W_{r}}{W_{0}}$$

where *W*_0_ is the weight of dried sample (g); *W_t_* is the weight of sample after rehydration (g).

### 2.8. Total Phenolic Content (TPC)

The total phenolic content (TPC) of the dried green pea samples was determined using a modified Folin–Ciocalteu method [36]. For the analysis, 0.5 g of finely ground dried pea sample was mixed with 10 mL of 70% ethanol solution in a 15 mL centrifuge tube and extracted in an ultrasonic extractor at 30 °C for 40 min. The extract was then centrifuged at 8000 r/min for 30 min, and the supernatant was collected for TPC measurement. A total of 1.0 mL of the extract was added to a 10 mL stoppered test tube with 1 mL of Folin–Ciocalteu reagent, followed by 2 mL of a 7.5% sodium carbonate solution. The solution was diluted to volume with distilled water and allowed to react in the dark at room temperature for 1 h. The absorbance was measured at 765 nm using a UV/VIS spectrophotometer (754, Shanghai Jinghua Technology Instrument Co., Ltd., Shanghai, China), with a blank prepared by using the extraction solvent instead of the extract. A standard curve was prepared using gallic acid, and the TPC of the dried pea samples was calculated and expressed as milligrams of gallic acid equivalents (GAE) per gram of dry weight (mg GAE/g DW).

### 2.9. DPPH Radical Scavenging Activity

The DPPH radical scavenging activity of dried green peas was assessed using a modified version of the method described in reference [37]. First, 0.2 g of finely ground dried pea sample was extracted with 10 mL of 70% ethanol in an ultrasonic extractor at 30 °C for 40 min. After extraction, the mixture was centrifuged at 8000 r/min for 10 min, and the supernatant was collected for analysis. A total of 2 mL of 0.1 mM DPPH solution was transferred into a test tube, followed by the addition of 1 mL of anhydrous ethanol. The mixture was thoroughly mixed, and the absorbance of this control solution was measured at 517 nm to obtain *A_control_*. Separately, 200 μL of the sample extract was added to another test tube with 800 μL of anhydrous ethanol and 2 mL of DPPH solution, and this sample mixture was also thoroughly mixed. Both the control and sample solutions were then incubated in the dark at 25 °C for 30 min. The absorbance of the sample solution was subsequently measured at 517 nm to obtain *A_sample_*. The DPPH radical scavenging activity was calculated using Equation (8):

(8) $$\mathrm{DPPH}\;\mathrm{free}\;\mathrm{radical}\;\mathrm{scavenging}\;\mathrm{activity}\;(\%)=\frac{A_{\mathrm{control}}-A_{sample}}{A_{\mathrm{sample}}}\text{×}100\%$$

where *A_control_* is the absorbance of the DPPH solution without extract, and *A_sample_* is the absorbance of the DPPH solution with extract. Results were expressed as the percentage of radical scavenging activity, indicating the antioxidant capacity of the dried pea samples.

### 2.10. Microstructure Observation

Scanning electron microscopy (S-3400 N, Hitachi Ltd., Tokyo, Japan) was used to examine the microstructure of untreated and pretreated dried green pea seed coats at an accelerating voltage of 15 kV under a vacuum. Samples were cut into 3 mm × 3 mm cross-sectional blocks, and microstructural images were captured at magnifications of 100×, 500×, and 1000× following sample preparation and gold coating.

### 2.11. Statistical Analysis

The experiment was conducted in triplicate unless otherwise noted, and data are presented as mean ± standard deviation. Experimental data were analyzed using Origin v.2022 (OriginLab Corp., Northampton, MA, USA), while statistical analyses were performed with SPSS v.26.0 (SPSS Inc., Chicago, IL, USA). One-way analysis of variance (ANOVA) followed by Duncan’s test (*p* < 0.05) was used to assess significant differences between groups. Correlations between parameters were evaluated using Spearman’s correlation test.

## 3. Results and Discussion

### 3.1. Drying Curves

**Figure 2:**
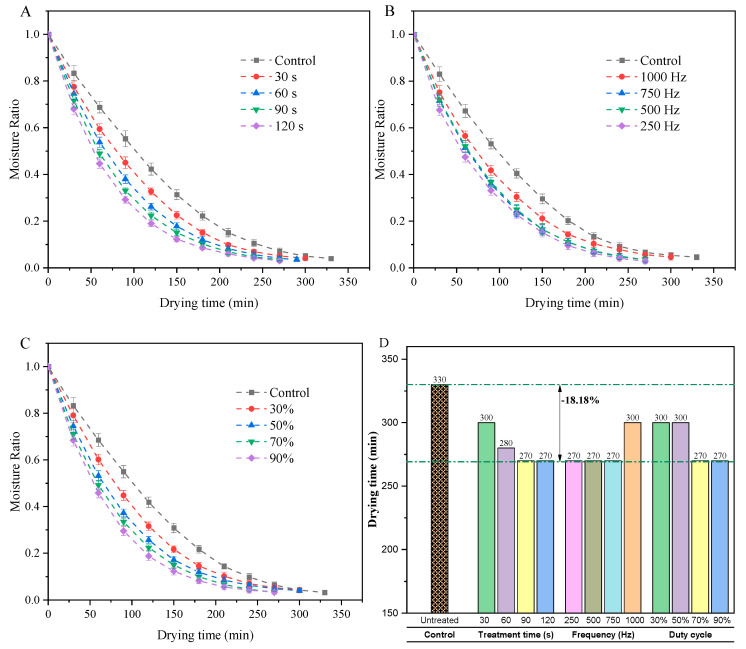
Drying curves of green peas under cold plasma (CP) treatments with varying parameters. (**A**): Effect of CP treatment duration (30–120 s, frequency: 750 Hz, duty cycle: 70%); (**B**): effect of CP excitation frequency (250–750 Hz, duration: 90 s, duty cycle: 70%); (**C**): effect of CP duty cycle (30–90%, frequency: 750 Hz, duration: 90 s); (**D**): total drying times of green peas under different CP treatment conditions.

The drying curves of green peas treated with cold plasma (CP) at various times, frequencies, and duty cycles are shown in Figure 2. The moisture ratio (MR) of both untreated samples (control) and CP-pretreated green peas followed a similar trend during the drying process. The MR decreased significantly in the initial stage, gradually declined as drying progressed, and eventually stabilized, approaching a constant value. CP-treated green peas exhibit a significantly shorter drying time compared to the untreated samples, demonstrating a notable improvement in drying efficiency. The cuticle and wax layers on the surface of green peas possess natural hydrophobic properties and protective functions that impede moisture migration during the drying process [38,39]. Etching by reactive species in the plasma disrupts these protective layers, leading to the structural etching of the cuticle and wax layers [28].

This etching process creates microscopic pits and pores on the surface of the green peas, increasing their specific surface area and thereby facilitating moisture diffusion and evaporation [8,21]. Additionally, reactive oxygen species (such as oxygen radicals and ozone) generated in the plasma interact with the green pea surface, forming oxygen-containing functional groups, such as hydroxyl and carboxyl groups [40]. These functional groups enhance the surface’s affinity for water molecules, enabling easier adsorption and faster diffusion of moisture [11,41,42].

As shown in Figure 2A, when the CP treatment duration increased from 30 s to 120 s (frequency: 750 Hz, duty cycle: 70%), the times required for green peas to reach the target moisture content were 300, 280, 270, and 270 min, respectively. This trend suggests that the drying time of green peas decreases with increased CP treatment duration up to 90 s but does not shorten further beyond 90 s (with a steady drying time of 270 min). This phenomenon may be attributed to the surface modification effects of CP treatment approaching a saturation point at 90 s. Additional CP exposure likely does not significantly enhance the extent of etching or the formation of hydrophilic functional groups.

When the excitation frequency of CP treatment increases from 250 Hz to 750 Hz (time: 90 s, duty cycle: 70%), the drying time of green peas remains constant at 270 min. However, at the highest frequency of 1000 Hz, the required drying time increases to 300 min (Figure 2B). From a theoretical perspective, at lower frequencies (250–750 Hz), ions in the plasma field acquire sufficient energy due to the longer duration of each oscillation cycle, enabling effective surface etching and modification. These changes enhance the surface’s hydrophilicity and permeability, facilitating moisture diffusion and maintaining shorter drying times. Conversely, at 1000 Hz, the rapid oscillations in the plasma field reduce the time available for ion acceleration, resulting in lower ion energy and less effective surface etching. This weaker surface modification decreases hydrophilicity and permeability, thereby slowing moisture diffusion and prolonging the drying time. These findings align with the study by Loureiro et al. [43], who observed similar effects of CP treatment on the drying process of tucuma (*Astrocaryum aculeatum*).

As the CP duty cycle increases from 30% to 90% (at 750 Hz for 90 s), the drying time for green peas remains at 300 min for 30% and 50% duty cycles but decreases to 270 min for 70% and 90% duty cycles. The duty cycle in plasma processing, defined as the ratio of the time the plasma is active to the total cycle time [44,45], plays a critical role in determining the intensity of plasma–surface interactions. At higher duty cycles (70% and 90%), the plasma exposure is more continuous, allowing for prolonged interaction between reactive species, such as oxygen radicals, and the surface. This extended exposure promotes the formation of microstructures, increases surface hydrophilicity, and enhances moisture diffusion. In contrast, at lower duty cycles (30% and 50%), the reduced active plasma exposure limits the extent of surface interaction, resulting in less effective modification and slower moisture release.

The findings demonstrate that CP pretreatment enhances the drying efficiency of green peas, achieving a drying time reduction of up to 18.18% compared to untreated samples (Figure 2D). However, the results also reveal that further increases in CP treatment duration or etching intensity do not consistently lead to better drying performance. These variations may reflect complex interactions between plasma-induced surface modifications and the physical properties of green peas, indicating that precise control of CP parameters is essential for optimizing drying efficiency without compromising nutritional quality.

### 3.2. Moisture Effective Diffusivity Coefficients

The linear regression equations for the natural logarithm of the moisture ratio (*lnMR*) versus drying time (*t*) of green peas under various CP pretreatment conditions (treatment time, frequency, and duty cycle) are presented in Table 1. The corresponding moisture effective diffusivity coefficients (*D_eff_*), calculated from these equations, are also included. The data show that the coefficients of determination (*R^2^*) for the linear regression equations of *lnMR* versus drying time are all above 0.99, indicating that Fick’s second law accurately describes the moisture diffusion behavior of peas during drying. The *D_eff_* values for both untreated and CP-pretreated peas range from 5.9629 to 9.9172 × 10^−10^ m^2^·s^−1^, which is consistent with the typical *D_eff_* range reported for hot-air drying of fruits and vegetables [46,47].

As the duration of CP treatment increased (from 30 s to 60, 90, and 120 s), the *D_eff_* of green peas progressively improved. Compared to untreated peas, *D_eff_* increased by approximately 19.39%, 38.12%, 54.93%, and 63.91%, respectively, indicating that longer CP treatment durations have a more significant effect on enhancing *D_eff_*. When the treatment frequency increased from 250 Hz to 500 Hz, 750 Hz, and 1000 Hz (treatment time: 90 s, duty cycle: 70%), *D_eff_* increased by approximately 66.31%, 53.20%, 54.93%, and 7.72%, respectively, compared to untreated peas. This demonstrates that frequency increases significantly enhance *D_eff_* within a certain range, though the effect diminishes at higher frequencies (1000 Hz). Similarly, as the duty cycle increased from 30% to 50%, 70%, and 90%, *D_eff_* significantly improved, with increases of approximately 17.61%, 26.89%, 54.93%, and 61.24%, respectively. This suggests that higher duty cycles can substantially enhance *D_eff_*. These findings on moisture effective diffusivity provide insights into the reasons for differences in drying times from the perspective of moisture diffusion.

The results demonstrate that plasma pretreatment parameters—treatment time, frequency, and duty cycle—significantly influence the *D_eff_* of green peas. Extending the treatment duration and increasing the duty cycle can substantially enhance *D_eff_*, while optimizing the frequency requires careful consideration within a specific range to avoid diminished effects at excessively high frequencies. Selecting appropriate treatment parameters can effectively improve *D_eff_*, providing valuable insights into the moisture migration process during practical drying operations.

### 3.3. Color

Table 2 presents the color parameters of dried green peas subjected to various CP treatments. The data reveal that, regardless of the specific CP treatment parameters, CP-pretreated green peas exhibit a significant decrease in L* and a* values, an increase in b* values, and a notable reduction in color difference (Δ*E*) compared to untreated peas. This suggests that CP-treated peas, after drying, display brighter colors, with more pronounced green and yellow hues, closely resembling the appearance of fresh samples.

Research indicates that CP treatment can inhibit the activity of polyphenol oxidase (PPO) and peroxidase (POD) in peas, thereby mitigating enzymatic browning reactions [48,49]. Additionally, CP pretreatment shortens the drying time of samples, reducing their prolonged exposure to high temperatures and oxygen during the drying process [20]. By limiting these conditions, CP treatment preserves chlorophyll-related greenness and minimizes browning reactions [50]. Consequently, CP-treated peas demonstrate enhanced color stability, reduced chlorophyll degradation, and minimized browning compared to untreated samples, ultimately improving the product’s overall color quality.

Specifically, as shown in Table 2, increasing the treatment time from 30 s to 90 s resulted in a higher L* value (59.93 ± 2.32) and a lower Δ*E* value (9.41 ± 0.62). This indicates a significant improvement in brightness and a reduction in browning, producing a color closer to that of fresh peas. However, extending the treatment time to 120 s led to a noticeable increase in the Δ*E* value. Frequency also plays a crucial role in determining color outcomes. At a lower frequency (250 Hz), the improvement in the L* value was minimal, and the Δ*E* value remained high (13.87 ± 1.67), indicating a limited effect on color enhancement. In contrast, at 750 Hz, the peas showed the best results, with the highest L* value and the lowest Δ*E* value, making this frequency the most effective for maintaining color quality. Conversely, frequencies above 750 Hz (e.g., 1000 Hz) slightly diminished the color improvement, with a slight increase in the Δ*E* value, possibly due to insufficient treatment intensity at excessively high frequencies. The duty cycle also influenced the color significantly. As the duty cycle increased from 30% to 70%, the L* and b* values gradually rose, while the Δ*E* value progressively decreased. However, raising the duty cycle to 90% slightly enhanced brightness but increased the a* value, leading to a slightly higher Δ*E* value. This could be attributed to the adverse effects of excessive treatment by high-energy particles. Therefore, a treatment time of 90 s, a frequency of 750 Hz, and a duty cycle of 70% were the most effective parameters for enhancing brightness, reducing color difference, and suppressing browning, resulting in dried green peas with optimal color quality.

### 3.4. Rehydration Ratio

The rehydration ratio (RR) is considered a key quality attribute of dried products [51], as most dried products are typically rehydrated before consumption. Table 2 shows the effect of different CP pretreatment times, frequencies, and duty cycles on the RR of green peas. As seen in the table, regardless of the CP treatment conditions used in this experiment, the RR values (2.28–3.29) of CP-treated green peas were higher than that of untreated green peas (2.15 ± 0.05). This indicates that CP treatment significantly enhances the rehydration capacity of dried green peas, accelerating the rehydration process.

It has been reported that CP pretreatment also improved the rehydration ratio of apple slices (up to 21.63%) [22]; tucuma samples treated with cold plasma showed a higher rehydration rate than untreated samples [43]; and CP pretreatment increased the rehydration ratio of goji berries by 7–16% [25]. Furthermore, sliding arc plasma pretreatment positively affected the rehydration rate of grapes [27]. The beneficial effect may be attributed to the etching effect of CP, which corrodes the cuticle on the surface of green peas, forming a microporous structure [38,52], thus increasing the specific surface area and facilitating faster and more efficient water absorption during the rehydration process. Additionally, CP treatment enhances surface hydrophilicity by introducing polar functional groups (e.g., hydroxyl or carboxyl groups), further increasing the affinity of the pea surface for water molecules [40].

Table 2 also shows that CP treatment time, frequency, and duty cycle affect the RR. Specifically, extending the treatment time to 90 s, lowering the frequency to below 750 Hz, and increasing the duty cycle can effectively improve the RR. This improvement can be attributed to the generation of reactive species during plasma treatment, which modifies the sample surface by enhancing porosity and increasing water absorption capacity. However, it is also noteworthy that excessive treatment conditions, such as prolonged treatment times (120 s) or low-frequency treatments (250 Hz), lead to a decrease in RR. This may be due to excessive etching, which compromises the structural integrity of the pea surface, reducing pore functionality or causing the collapse of the porous network. Therefore, this suggests that moderate CP treatment strikes a balance between surface modification and structural preservation, achieving optimal rehydration performance.

### 3.5. Total Polyphenol Content

**Figure 3:**
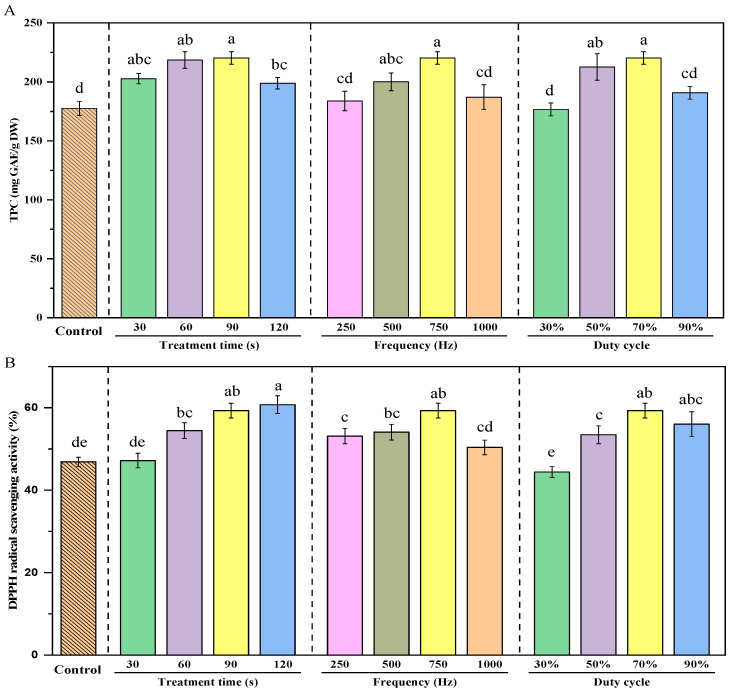
Total polyphenol content (TPC) and DPPH free radical scavenging activity of dried green peas. (**A**): TPC of green peas under different cold plasma treatment times, frequencies, and duty cycles; (**B**): DPPH free radical scavenging activity of green peas under different cold plasma treatment times, frequencies, and duty cycles. Note: In the treatment time group, the frequency is fixed at 750 Hz with a duty cycle of 70%; in the frequency group, the treatment time is fixed at 90 s with a duty cycle of 70%; in the duty cycle group, the frequency is fixed at 750 Hz with a treatment time of 90 s. Different letters in the bar chart indicate statistically significant differences (*p* < 0.05).

The total polyphenol content (TPC) of green peas was significantly influenced by the parameters of cold plasma treatment, including treatment time, frequency, and duty cycle (Figure 3A). All CP-treated samples exhibited significantly higher TPC compared to the control group (*p* < 0.05). In terms of treatment time, the TPC increased as the duration extended, reaching a maximum at 90 s, followed by a slight decline at 120 s. This suggests that prolonged exposure may lead to polyphenol degradation [25,27]. For frequency, the TPC displayed a parabolic trend, peaking at 750 Hz. Lower frequencies (250 Hz) and higher frequencies (1000 Hz) resulted in a reduced TPC, indicating that an optimal frequency range is crucial for maximizing polyphenol enhancement. Similarly, the duty cycle exhibited a significant influence, with the TPC reaching its maximum at 70% and decreasing at 90%. The highest TPC (~220.31 mg GAE/g DW) was achieved under the optimal cold plasma treatment conditions of a 90 s treatment time, 750 Hz frequency, and 70% duty cycle. This represents a significant increase of approximately 24.06% compared to the control group, highlighting the efficacy of cold plasma treatment in enhancing the polyphenol content in green peas.

The beneficial effects of CP treatment in increasing the TPC have been documented by several researchers. For instance, CP treatment increased the TPC of goldenberries by 52.31% compared to untreated convection-dried samples [11]. Similarly, cold plasma treatment enhanced the TPC of jujube by 12% [21]. The TPC of CP-pretreated saffron was significantly higher than that of untreated groups [53]. Additionally, CP pretreatment improved grape quality, with TPC retention ranging from 3.06% to 30.53% higher than that of untreated grapes [27].

Two possible mechanisms may explain these effects. First, CP treatment disrupts plant cell structures, facilitating the release of intracellular polyphenols. Polyphenolic compounds are typically stored in specific cellular compartments, such as vacuoles or the cell wall. Plasma-induced structural damage makes these compounds more accessible for extraction [54]. Second, cold plasma inhibits the activity of oxidative enzymes such as polyphenol oxidase (PPO) and peroxidase (POD), which are key contributors to polyphenol degradation. By reducing enzymatic activity, CP treatment helps preserve higher polyphenol levels in treated samples compared to controls.

### 3.6. DPPH Free Radical Scavenging Activity

The DPPH free radical scavenging activity of dried green peas treated with CP and untreated (control) is shown in Figure 3B. The DPPH radical scavenging activity of green peas was significantly enhanced by cold plasma treatment compared to the untreated control group, which showed the lowest activity. As the treatment time increased from 30 s to 120 s, scavenging activity progressively improved, with 120 s achieving the highest activity. Frequency exhibited a nonlinear effect: scavenging activity increased from 250 Hz to 750 Hz but declined at 1000 Hz, with 750 Hz being the most effective. Similarly, the duty cycle showed a positive correlation with scavenging activity, as higher duty cycles (50%, 70%, and 90%) significantly enhanced the antioxidant response compared to 30%, though the improvement diminished at 90%, suggesting saturation.

From the analysis, the optimal conditions for maximizing DPPH radical scavenging activity were identified as a treatment time of 90–120 s, a frequency range of 750 Hz, and a 70% duty cycle. Under these optimized CP treatment conditions, the DPPH radical scavenging activity of the treated samples increased by approximately 29.64% compared to the untreated samples. This significant improvement is likely due to the localized rupture of cell walls or membranes caused by cold plasma, which promotes the release of antioxidants, making them more readily available for antioxidant reactions [55]. Furthermore, CP treatment can shorten the time required for the subsequent drying process, thus reducing the thermal degradation loss of antioxidant compounds (such as polyphenols and vitamin C) [27,56]. Therefore, cold plasma technology, as a non-thermal pretreatment method, significantly enhances antioxidant activity and provides valuable technological support for food processing.

### 3.7. Microstructure

**Figure 4:**
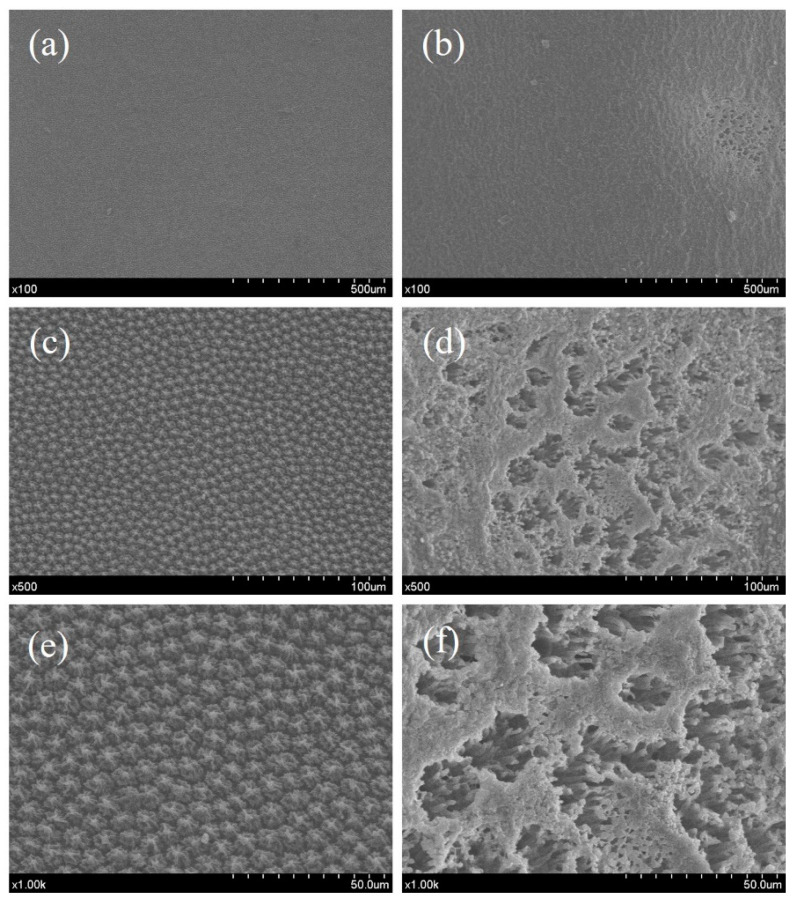
Microstructure of green pea pretreated by cold plasma and control sample at different magnifications (100×, 500×, and 1000×). (**a**,**c**,**e**), Untreated control sample; (**b**,**d**,**f**), treated by cold plasma condition (90 s treatment time, 750 Hz frequency, and 70% duty cycle).

The microstructure of green pea epidermis, as observed under SEM at varying magnifications (100×, 500×, and 1000×), revealed significant alterations following CP treatment (90 s treatment time, 750 Hz frequency, and 70% duty cycle) compared to the untreated control sample (Figure 4). The SEM images reveal that CP treatment caused significant changes to the microstructure of green pea epidermis compared to the untreated control samples. The control samples exhibited a smooth, compact, and orderly surface structure, indicating intact and undisturbed epidermal cell walls (Figure 4a,c,e). In contrast, the CP-treated samples showed pronounced surface roughness, characterized by pore formation, disorganized cellular patterns, and fissures (Figure 4b,d,f).

CP treatment disrupts the integrity of the epidermal cell wall, likely due to the interaction of reactive oxygen species (ROS) and reactive nitrogen species (RNS) with the surface, leading to oxidative degradation of key cell wall components, such as cellulose, hemicellulose, and pectin. Additionally, the etching effect of CP treatment creates surface pores and cracks [57,58], further altering the microstructure of the epidermis. This etching effect, combined with oxidative damage, alters the microstructure of the green pea epidermis, where reactive species interact with the cell wall materials, inducing both physical and chemical changes [8,42]. Namjoo et al. [59] observed that CP pretreatment altered the surface microstructure of cumin seeds by generating micropores. Similarly, Yuan et al. [60] reported that CP pretreatment induced a porous structure in jujube slices. Huang et al. [61] also noted that plasma treatment caused the overall dissociation of the waxy cuticle in white grapes and observed the presence of cracks.

These structural alterations can partly explain the increased moisture diffusion rate, improved rehydration, and changes in nutrient content observed in this study. These findings contribute to the growing understanding of how cold plasma influences plant material surfaces and how its microstructural changes affect downstream properties, such as moisture migration, texture, hydration, and the bioavailability of nutrients.

## 4. Conclusions

In this study, we investigated the effects of CP pretreatment parameters—treatment time, frequency, and duty cycle—on the drying kinetics and quality attributes of green peas. CP treatment reduced drying time by up to 18.18%, achieving the shortest drying duration of 270 min. The moisture effective diffusivity (*D_eff_*) increased by up to 66.31%, indicating significantly enhanced moisture migration efficiency in CP-pretreated samples compared to untreated samples. Moreover, optimal CP pretreatment conditions (90 s treatment time, 750 Hz frequency, 70% duty cycle) preserved color quality with a minimal total color difference (Δ*E*) of 9.41, improved the rehydration ratio (RR) to 3.29, enhanced the total polyphenol content (TPC) by 24.06%, and increased DPPH free radical scavenging activity by 29.64%. Scanning electron microscopy (SEM) revealed significant surface modifications in CP-pretreated green peas, including the formation of pores and fissures, which, to some extent, explained the enhanced moisture diffusion, improved drying efficiency, and increased rehydration ratio observed in the study.

These findings provide valuable insights into the application of CP as an innovative, non-thermal pretreatment method for improving drying efficiency and product quality in agricultural processing, which could have applications in food preservation and sustainable processing technologies. However, further research is needed to address the challenges of implementing continuous CP processing and to evaluate its efficiency and cost-effectiveness in large-scale industrial applications. Future studies should focus on optimizing CP system design for continuous operation and assessing its feasibility for commercial food processing, which will be critical for enhancing the practicality and economic viability of this technology.

## Figures and Tables

**Table 1:** Moisture effective diffusion coefficients of green peas under different cold plasma treatment times, frequencies, and duty cycles.

Number	Pretreatment Time/s	Frequency /Hz	Duty Cycle	Linear Regression Equation	*R* ^2^	*D_eff_* /10^−10^ m^2^·s^−1^
1	Control			ln*MR* = −1.7164 × 10^−4^ *t* + 0.2263	0.9880	5.9629
2	30	750	70%	ln*MR* = −1.8754 × 10^−4^ *t* + 0.1211	0.9948	7.1191
3	60	750	70%	ln*MR* = −2.0244 × 10^−4^ *t* + 0.0756	0.9988	8.2956
4	90	750	70%	ln*MR* = −2.1286 × 10^−4^ *t* + 0.2933	0.9995	9.2386
5	120	750	70%	ln*MR* = −2.1966 × 10^−4^ *t* − 0.0349	0.9980	9.7743
6	90	250	70%	ln*MR* = −2.2131 × 10^−4^ *t* + 0.3800	0.9986	9.9172
7	90	500	70%	ln*MR* = −2.1243 × 10^−4^ *t* + 0.0799	0.9980	9.1355
8	90	1000	70%	ln*MR* = −1.7805 × 10^−4^ *t* + 0.0387	0.9978	6.4232
9	90	750	30%	ln*MR* = −1.8097 × 10^−4^ *t* + 0.2265	0.9964	7.0132
10	90	750	50%	ln*MR* = −2.1489 × 10^−4^ *t* + 0.0366	0.9994	7.5664
11	90	750	90%	ln*MR* = −2.1794 × 10^−4^ *t* − 0.0418	0.9945	9.6148

**Table 2:** Color parameter and rehydration ratio (RR) of dried green peas under different cold plasma treatment times, frequencies, and duty cycles.

Pretreatment Time/s	Frequency /Hz	Duty Cycle	L^*^	a^*^	b^*^	Δ*E*	Rehydration Ratio
Control			46.26 ± 1.48 ^f^	4.32 ± 0.53 ^ab^	20.71 ± 1.37 ^cd^	18.23 ± 1.38 ^a^	2.15 ± 0.05 ^g^
30	750	70%	51.64 ± 0.49 ^cde^	2.48 ± 0.16 ^de^	24.08 ± 1.35 ^ab^	11.74 ± 0.81 ^cd^	2.72 ± 0.069 ^cde^
60	750	70%	56.33 ± 1.49 ^ab^	3.74 ± 0.82 ^abcd^	22.04 ± 1.73 ^abcd^	11.31 ± 1.84 ^cd^	2.87 ± 0.05 ^cd^
90	750	70%	59.93 ± 2.32 ^a^	3.81 ± 0.28 ^abcd^	24.37 ± 1.00 ^a^	9.41 ± 0.62 ^d^	3.28 ± 0.06 ^ab^
120	750	70%	50.32 ± 2.31 ^ef^	4.82 ± 0.56 ^a^	21.09 ± 1.58 ^bcd^	15.50 ± 2.16 ^ab^	2.57 ± 0.07 ^def^
90	250	70%	50.65 ± 1.63 ^de^	2.07 ± 0.82 ^e^	21.36 ± 1.82 ^abcd^	13.87 ± 1.67 ^bc^	3.22 ± 0.06 ^ab^
90	500	70%	56.62 ± 2.78 ^ab^	3.76 ± 0.05 ^abcd^	22.15 ± 1.00 ^abcd^	11.33 ± 1.60 ^cd^	3.29 ± 0.27 ^a^
90	750	70%	59.93 ± 2.32 ^a^	3.81 ± 0.28 ^abcd^	24.37 ± 1.00 ^a^	9.41 ± 0.62 ^d^	3.28 ± 0.06 ^ab^
90	1000	70%	55.47 ± 1.16 ^bc^	3.96 ± 0.22 ^abc^	23.15 ± 0.48 ^abcd^	10.98 ± 0.51 ^cd^	2.98 ± 0.05 ^bc^
90	750	30%	49.36 ± 1.22 ^ef^	3.34 ± 1.03 ^bcde^	20.48 ± 0.88 ^d^	15.70 ± 1.40 ^ab^	2.28 ± 0.03 ^fg^
90	750	50%	54.81 ± 2.19 ^bcd^	2.66 ± 0.08 ^cde^	23.81 ± 0.73 ^abc^	10.21 ± 0.77 ^d^	2.49 ± 0.17 ^ef^
90	750	70%	59.93 ± 2.32 ^a^	3.81 ± 0.28 ^abcd^	24.37 ± 1.00 ^a^	9.41 ± 0.62 ^d^	3.28 ± 0.06 ^ab^
90	750	90%	59.88 ± 1.28 ^a^	4.35 ± 0.81 ^ab^	23.47 ± 1.40 ^abcd^	10.22 ± 1.02 ^d^	3.29 ± 0.07 ^a^

Note: Data are expressed as the average ± standard deviation. Values in the same column having the different letters (a–g) for each parameter are significantly different (*p* < 0.05).

## Data Availability

The original contributions presented in the study are included in the article; further inquiries can be directed to the corresponding author.
